# Directed Differentiation of Human Induced Pluripotent Stem Cells into Dendritic Cells Displaying Tolerogenic Properties and Resembling the CD141^+^ Subset

**DOI:** 10.3389/fimmu.2017.01935

**Published:** 2018-01-08

**Authors:** Patty Sachamitr, Alison J. Leishman, Timothy J. Davies, Paul J. Fairchild

**Affiliations:** ^1^Sir William Dunn School of Pathology, University of Oxford, Oxford, United Kingdom

**Keywords:** induced pluripotent stem cell, dendritic cell, regulatory T cell, directed differentiation, tolerance, CD141

## Abstract

The advent of induced pluripotent stem cells (iPSCs) has begun to revolutionize cell therapy by providing a convenient source of rare cell types not normally available from patients in sufficient numbers for therapeutic purposes. In particular, the development of protocols for the differentiation of populations of leukocytes as diverse as naïve T cells, macrophages, and natural killer cells provides opportunities for their scale-up and quality control prior to administration. One population of leukocytes whose therapeutic potential has yet to be explored is the subset of conventional dendritic cells (DCs) defined by their surface expression of CD141. While these cells stimulate cytotoxic T cells in response to inflammation through the cross-presentation of viral and tumor-associated antigens in an MHC class I-restricted manner, under steady-state conditions CD141^+^ DCs resident in interstitial tissues are focused on the maintenance of homeostasis through the induction of tolerance to local antigens. Here, we describe protocols for the directed differentiation of human iPSCs into a mixed population of CD11c^+^ DCs through the spontaneous formation of embryoid bodies and exposure to a cocktail of growth factors, the scheduled withdrawal of which serves to guide the process of differentiation. Furthermore, we describe the enrichment of DCs expressing CD141 through depletion of CD1c^+^ cells, thereby obtaining a population of “untouched” DCs unaffected by cross-linking of surface CD141. The resulting cells display characteristic phagocytic and endocytic capacity and acquire an immunostimulatory phenotype following exposure to inflammatory cytokines and toll-like receptor agonists. Nevertheless, under steady-state conditions, these cells share some of the tolerogenic properties of tissue-resident CD141^+^ DCs, which may be further reinforced by exposure to a range of pharmacological agents including interleukin-10, rapamycin, dexamethasone, and 1α,25-dihydoxyvitamin D_3_. Our protocols therefore provide access to a novel source of DCs analogous to the CD141^+^ subset under steady-state conditions *in vivo* and may, therefore, find utility in the treatment of a range of disease states requiring the establishment of immunological tolerance.

## Introduction

Through their unrivaled capacity for antigen processing and presentation, dendritic cells (DCs) are uniquely equipped to engage naïve T cells in dialog, implicating them in the genesis of all immune responses (1). As such, DCs are responsible for defining the outcome of antigen recognition, either ensuring robust immunity to a microbial challenge or pacifying deleterious autoimmune responses through the induction and maintenance of immunological tolerance. Which of these diametrically opposed outcomes prevails depends primarily on the context in which antigen presentation by DCs occurs, steady-state conditions promoting the maintenance of tolerance, while ongoing inflammation favors immunity (1). These properties have made DCs attractive therapeutic agents for intervening in the progression of an immune response, inspiring numerous clinical trials for vaccination to poorly immunogenic tumor associated antigens (TAAs) as the basis for cancer immunotherapy (2). Furthermore, the clinical application of DCs has recently extended beyond vaccination to the induction of antigen-specific tolerance for the treatment of autoimmune diseases as diverse as diabetes (3, 4), multiple sclerosis (5), and rheumatoid arthritis (6, 7) as well as the prevention of allograft rejection (8, 9). While these trials have shown a good safety profile (3), they have yet to demonstrate significant efficacy: for instance, recent analyses of over 54 clinical trials for melanoma revealed objective response rates of less than 10% (10).

Such disappointing outcomes may be attributed in part to the identity of the DCs employed in clinical trials which, for pragmatic reasons, are most commonly differentiated *in vitro* from the patient’s own peripheral blood monocytes which may be subsequently matured by exposure to inflammatory cytokines or treated with a range of pharmacological agents such as interleukin (IL) 10, dexamethasone, rapamycin, and 1α,25-dihydroxyvitamin D_3_ (VD_3_), widely demonstrated to restrain their immunogenicity and render them more tolerogenic (11). Although ease of access confers a significant advantage on monocyte-derived DCs (moDCs), they are known to exhibit substantial donor-to-donor variation, which may be exacerbated by exposure of patients to long-term chemotherapy or immune suppression. Furthermore, moDCs display poor capacity for the cross-presentation of soluble or cellular antigens to MHC class I-restricted CD8^+^ T cells. Antigen cross-presentation is not only a requirement for induction of the cytotoxic T lymphocyte (CTL) responses essential for the clearance of an established tumor (2) but has also been strongly implicated in the maintenance of “cross-tolerance” among CD8^+^ T cells under steady-state conditions (12). The use of alternative subsets of DCs with proven capacity for the cross-presentation of soluble and cellular antigens may, therefore, provide a rational alternative to the widespread use of moDCs for immunotherapy.

In the human, conventional DC (cDC) belong to two distinct subsets, identified by their surface expression of CD1c or CD141. These subsets derive from a common progenitor which fails to give rise to monocytes or plasmacytoid DCs, formally distinguishing them from either lineage (13). CD141^+^ DCs were recently shown to exhibit superior capacity for antigen cross-presentation (14–17). Furthermore, they may be defined by their co-expression of toll-like receptor (TLR) 3, Clec9A and the chemokine receptor, XCR1 and have been shown to be critical for eliciting responses to tumor and viral antigens without requiring either direct infection or endogenous expression of TAAs (18). To perform such a function, CD141^+^ DCs are highly endocytic and phagocytic, permitting their efficient acquisition of both soluble and cellular antigens (19). Through cross-presentation of acquired antigen in concert with IL-12 secretion, CD141^+^ DCs induce the activation of CTL to which they are attracted by virtue of their secretion of XCL1, the only known ligand of the XCR1 receptor (20). While such responses are commonly initiated in the secondary lymphoid organs in response to inflammation, CD141^+^ DCs have also been found in non-lymphoid tissues including the skin, lung, kidney, and liver (21, 22) where they constitute the most abundant subset (18). In these anatomical locations, CD141^+^ DCs have been shown to perform an essential regulatory role in the steady-state in order to maintain tissue homeostasis. In the skin, for example, CD141^+^ DCs have been shown to express a distinctive CD14^+^ CD1a^−^ CD207^−^ phenotype and constitutively secrete the anti-inflammatory cytokine IL-10 (23). Their capacity for expansion of CD4^+^ regulatory T cells (Tregs) *in situ* was shown to reinforce tissue homeostasis and actively antagonize local inflammatory responses (23). The tolerogenicity of tissue-resident CD141^+^ DCs and their proven capacity for antigen cross-presentation may, therefore, provide a compelling rationale for their use in immunotherapies aimed at intervening in the progression of deleterious immune responses. Nevertheless, such plans have so far been confounded by the complexities of their distribution *in vivo*.

Although CD141^+^ DCs may be isolated from peripheral blood, these cells are thought to represent immature precursors of their tissue-resident counterparts (21). Furthermore, they represent the smallest subset of DCs in the peripheral circulation, constituting 0.03% of mononuclear cells. Consequently, a single leukapheresis has been estimated to yield as few as 3 × 10^5^ cells following purification, posing a significant barrier to their downstream clinical application (24). Various strategies have sought to overcome these limitations: culture of human CD34^+^ hematopoietic progenitor cells with a cytokine cocktail supplemented with the aryl hydrocarbon receptor antagonist StemRegenin 1 (SR1) promoted the *ex vivo* expansion of CD141^+^ DCs but showed no specificity for this subset, resulting in the simultaneous expansion of both plasmacytoid and CD1c^+^ DCs (25). Using an alternative approach, Ding and colleagues showed that NOD/SCID mice humanized using hematopoietic stem cells purified from cord blood, responded to administration of FLT3-Ligand by the generation of large numbers of both CD1c^+^ and CD141^+^ DCs (24). Nevertheless, such an approach is impractical for the purposes of scale-up and is incompatible with the generation of autologous cells, essential for their application to the induction of tolerance. Furthermore, the administration of FLT3-Ligand to healthy volunteers as a way of accessing autologous material resulted in the preferential expansion of cells expressing CD1c (26). Given the potential therapeutic benefits of harnessing the immunoregulatory properties of steady-state CD141^+^ DCs, we have, therefore, sought to overcome their paucity in peripheral blood and difficulties in their expansion from precursors *ex vivo*, by directing their differentiation from established lines of pluripotent stem cells.

We have previously demonstrated the feasibility of differentiating populations of primary DCs from both mouse and human embryonic stem cells (ESCs) (27, 28), thereby offering access to potentially unlimited numbers of cells, amenable to quality control. The advent of induced pluripotency and the derivation of induced pluripotent stem cells (iPSCs) under cGMP conditions (29) has led various groups to adapt our protocols developed using ESCs to the differentiation of DCs from human iPSCs (30, 31): nevertheless, such populations of iPSC-derived DCs (ipDCs) appear to belong predominately to the CD1c^+^ subset (31). We have, therefore, recently optimized our protocols for use with patient-specific iPSCs and have reported the directed differentiation of DCs which, in addition to CD1c^+^ cells, include a substantial population of CD141^+^ DCs capable of the cross-presentation of melanoma antigens to naïve peripheral blood T cells (32). Given the tractability of iPSCs for genome editing, this novel source offers opportunities for the introduction of subtle phenotypic or functional traits that might enhance the utility of the downstream cell therapy product or gain insight into aspects of the biology of this rare and inaccessible cell type in humans (2). Indeed, Sontag and colleagues used CRISPR/Cas9-mediated genome editing of human iPSCs to generate a cell line deficient in the interferon regulatory factor 8 (IRF8) transcription factor and showed that differentiation of CD141^+^ DCs was selectively compromised, while production of the CD1c^+^ subset was largely preserved, providing clear evidence for a critical role for IRF8 in guiding lineage commitment toward the cross-presenting DC subset (33).

Given that iPSCs may serve as a source of autologous CD141^+^ DCs, we have investigated whether this novel population might also show utility in pacifying deleterious immune responses under steady-state conditions and whether a tolerogenic phenotype may be further reinforced by exposure to defined pharmacological agents. Here, we describe in detail the protocols we have developed for the *in vitro* differentiation of CD141^+^ DCs from human iPSCs, together with their subsequent enrichment and characterization. Their responsiveness to pharmacological agents known to reinforce the tolerogenic phenotype suggests new avenues for their use in the treatment of numerous disease states requiring the induction of immunological tolerance.

## Overview of the Procedure

**Figure 1:**
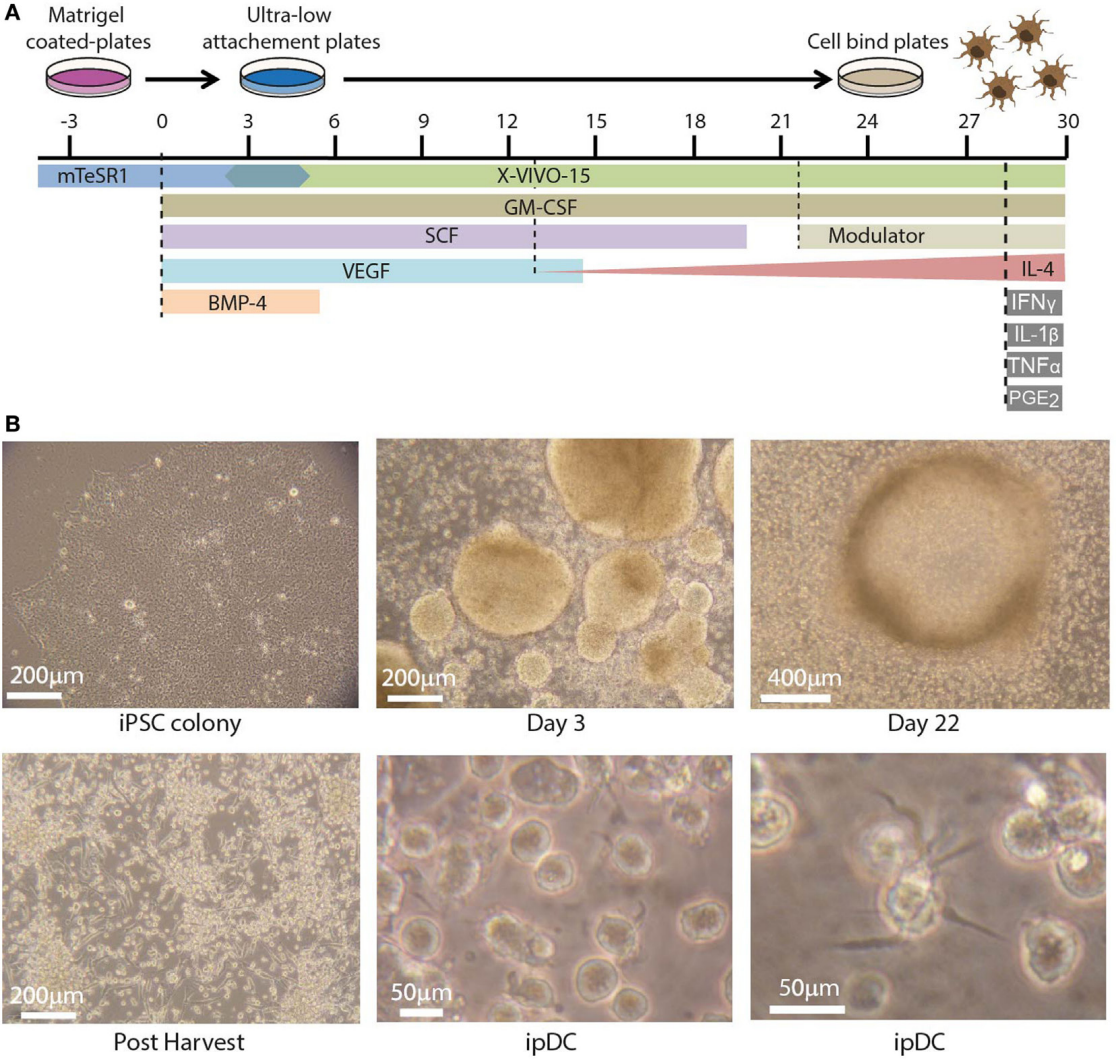
Differentiation of dendritic cells (DCs) from human induced pluripotent stem cells (iPSCs). **(A)** Timeline depicting the differentiation of human iPSCs into iPSC-derived DCs (ipDCs) using the protocol described for the addition and withdrawal of growth factors and cytokines. **(B)** Representative photomicrographs illustrating the morphology of colonies and individual cells during the differentiation process. Top left: colony of iPSCs cultured on matrigel in mTeSR-1 medium showing optimum morphology. Top center: early embryoid bodies on day 3 of culture on ultra-low attachment (ULA) plates in mTESR-1 supplemented with the full combination of growth factors. Top right: DC precursors at day 22 of culture accumulating around a single EB, from which they were originally released. Bottom left: DC precursors following harvesting onto cell bind plates to permit the adherence of macrophage-like cells. Bottom center and right: high magnification photomicrographs of fully differentiated ipDCs displaying characteristic DC morphology consisting of protrusions and veils of cytoplasm.

The use of human iPSCs as a novel source of potentially tolerogenic DCs expressing CD141 involves three distinct phases: (i) progressive differentiation of iPSCs *via* early mesoderm, through cells of the hematopoietic lineage, to committed DC precursors, (ii) modulation of the resulting ipDCs to reinforce their intrinsic tolerogenicity, and (iii) enrichment of the CD141^+^ subset. Figure 1A illustrates the timelines involved, together with the cytokine cocktail required to effect each stage of the differentiation pathway. In summary, iPSCs are expanded in culture during routine passage until the approximate number of cells required for differentiation is achieved. The iPSCs are harvested at 80–85% confluency (Figure 1B, top left) using 0.02% ethylenediaminetetraacetic acid (EDTA) and mechanical scraping to generate small colony fragments. These are subsequently plated in ultra-low attachment (ULA) plates in mTesR1 medium supplemented with recombinant human Granulocyte Macrophage Colony-Stimulating Factor (rhGM-CSF), Bone Morphogenetic Protein 4 (rhBMP-4), Stem Cell Factor (rhSCF), and Vascular Endothelial Growth Factor (rhVEGF). The cultures are fed routinely every 2–3 days with differentiation medium consisting of XVIVO-15 supplemented with the appropriate cytokines. Guided differentiation of the cells is achieved by the stepwise withdrawal of growth factors, starting with BMP-4 on day 5, VEGF on day 14, and SCF on day 19 of culture, leaving only GM-CSF to sustain DC precursors and immature DCs, whose terminal commitment is subsequently reinforced by the addition of IL-4.

Using this protocol, clusters of differentiating iPSCs may be observed on day 3 of culture, where they later give rise to structures known as embryoid bodies, which imperfectly recapitulate some of the earliest stages of embryogenesis (Figure 1B, top center). Around days 14–16, macrophage-like cells may be observed in the differentiation cultures. Upon appearance of these cells, the medium is supplemented with IL-4, the concentration of which increases progressively with each subsequent feed, starting with 25 ng/ml and increasing to a maximum concentration of 100 ng/ml. DC precursors and immature DCs accumulating around embryoid bodies (Figure 1B, top right) are normally harvested between days 21 and 26 by gentle pipetting and are subsequently plated on cell-bind plates in XVIVO-15 medium supplemented with GM-CSF and IL-4 alone (Figure 1B, bottom left). Under these conditions, any contaminating macrophages adhere strongly to the plastic, while immature DCs remain in suspension and are recognizable by their cytoplasmic protrusions (Figure 1B, bottom center), which tend to become more prominent over time (Figure 1B, bottom right).

**Figure 2:**
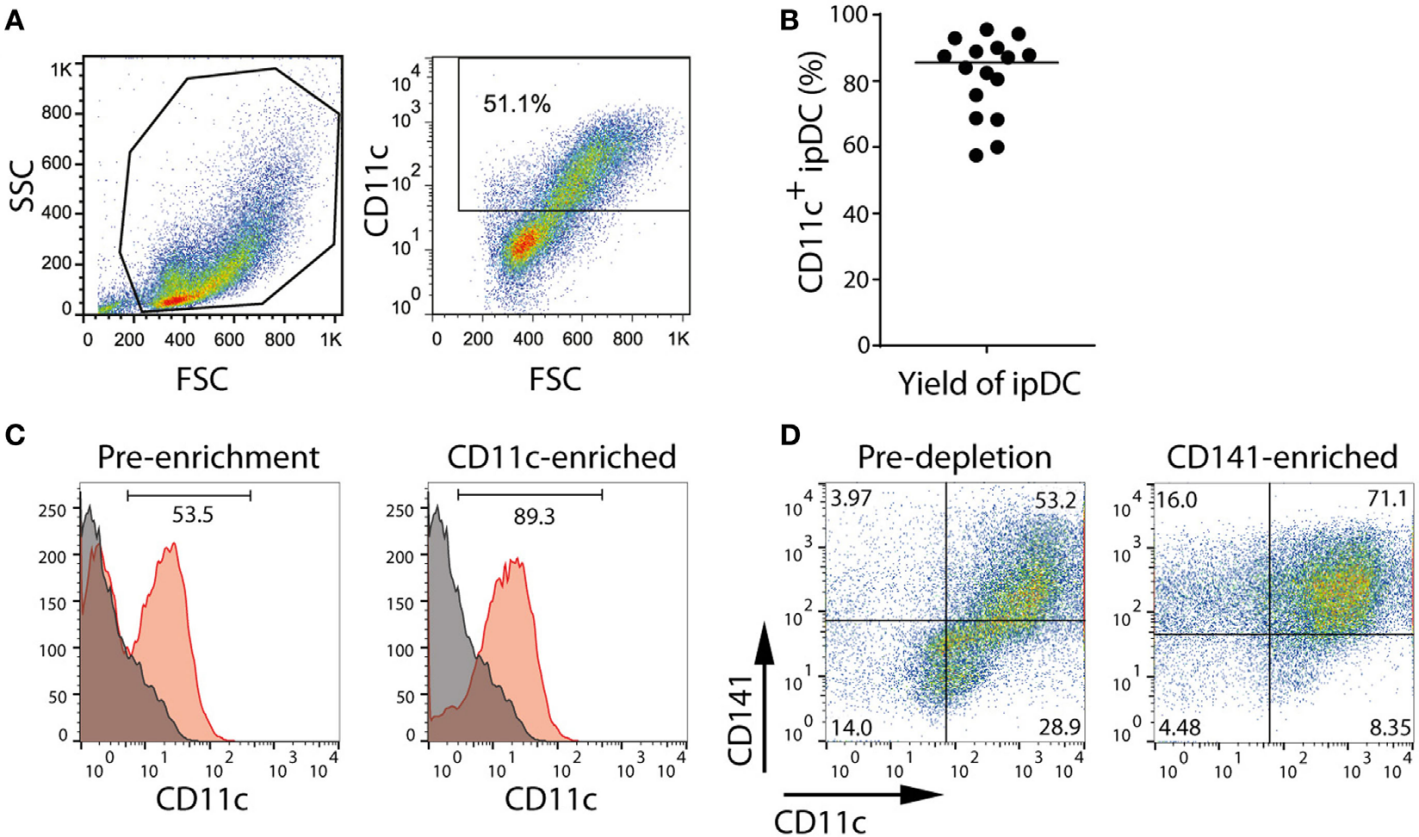
Purification of CD11c^+^ and CD141^+^ subsets of iPSC-derived DC (ipDCs) by magnetic bead separation. **(A)** FACS plot showing typical forward (FSC) and side scatter (SSC) of ipDCs obtained at the end of the differentiation procedure and the proportion of CD11c^+^ cells, which would normally suggest the need for their subsequent purification. **(B)** Percentage of CD11c^+^ cells obtained from 16 independent experiments. Each symbol represents an individual experiment, while the black line denotes the median (median = 85.55; SD = 11.94). **(C)** Enrichment of ipDCs from cultures containing lower proportions of CD11c^+^ cells: ipDCs were labeled with CD11c-biotin and purified using anti-biotin microbeads. CD11c expression is shown before and after purification. **(D)** Enrichment of “untouched” CD141^+^ ipDCs by depletion of CD1c^+^ cells using microbead separation. Co-expression of CD11c and CD141 is shown before and after enrichment, the quadrants being set according to non-specific staining with appropriately matched isotype controls. FACS plots are representative of three independent experiments.

In order to promote a tolerogenic phenotype, cultures of ipDCs are further supplemented with pharmacological agents previously proven to modulate the function of human moDCs, such as rapamycin, dexamethasone, VD_3_, or the anti-inflammatory cytokine IL-10 (11). While VD_3_ is added to cultures on days 0 and 3 after harvesting, dexamethasone, rapamycin, and IL-10 are added from day 3 onward. After 5 days, ipDCs may be additionally matured by exposure to a cocktail of inflammatory cytokines for 48 h, after which they may be harvested by gentle pipetting to resuspend the lightly adherent cells. The purity of cDCs obtained using our protocol may be determined as a function of CD11c expression using standard flow cytometry (Figure 2A). Although the proportion of CD11c^+^ cells may vary significantly between experiments, in our hands, the median percentage of cells expressing CD11c in 16 consecutive experiments was 85.5% (Figure 2B). However, in experiments yielding a purity below 60% (Figure 2A), cDCs may be enriched by labeling with monoclonal antibodies specific for CD11c and using magnetic bead separation techniques to isolate the labeled cells (Figure 2C).

Our attempts at purification of CD141^+^ ipDCs using protocols for their positive selection have been hampered by significant levels of cell death following cross-linking of CD141. To avoid this issue, CD141^+^ cells may be enriched by depletion of CD1c^+^ cDCs, with which they share a common progenitor (13). Removal of CD1c^+^ cells from cultures may likewise be achieved by separation using magnetic microbeads (Figure 2D). Protocols for each phase of the differentiation process outlined above, together with the reagents required, are described in detail below.

## Materials

### Cell Lines

Protocols for the maintenance and passage of existing iPSC lines are now well-established and have been reported in detail elsewhere (34). While we describe here the directed differentiation of CD141^+^ DCs from human iPSCs displaying some of the properties of the CD141^+^ subset described *in vivo*, the outcome of the protocols we describe is entirely dependent on the quality and status of the parent cell line: failure to maintain iPSCs under optimal conditions may have adverse effects on their subsequent differentiation capacity and may result in the progressive accumulation of mutations or karyotypic abnormalities for which the culture conditions may serve as a selection pressure. It is advisable, therefore, to submit cells for routine karyotyping and to replace cell cultures with an earlier passage, should abnormalities be observed that might threaten the integrity of the iPSC line. While our original experiments made use of the human iPSC line C15 derived from human dermal fibroblasts (35) (a kind gift from Lee Carpenter and Suzanne Watt, University of Oxford), the reproducibility of our data has since been verified using numerous human iPSC lines derived from both healthy volunteers and patients suffering from various disease states.

IMPORTANT! For all studies involving human subjects, ethical approval should first be sought from the appropriate ethical review body. In the United Kingdom, recruitment of patients requires approval from the local NHS National Research Ethics Service (NRES) and should only be conducted following the receipt of informed consent.

### Reagents

#### Cell Culture Media and Reagents

- mTeSR1 (STEMCELL Technologies, cat. no. 05850)
- X-VIVO-15 (Lonza, cat. no. BE04-418Q)
- Knockout DMEM (Life Technologies, cat. no. 10829-018)
- Dimethylsulfoxide (DMSO) (Sigma-Aldrich, cat. no. D2660-100ML)
- PBS (Life Technologies, cat. no. 10010-015)
- Non-essential amino acids (Sigma-Aldrich, cat. no. M7145)
- l-glutamine (PAA laboratories GmbH, cat. no. M11-004)
- Sodium pyruvate (PAA laboratories GmbH, S11-003)
- 2-Mercaptoethanol (2-ME) (Sigma-Aldrich, cat. no. M7522)
- Y-27632 (Calbiochem, cat. no. 688001)
- Bovine serum albumin (BSA) (Sigma-Aldrich, cat. no. A3311)
- Recombinant human serum albumin (HSA) (Sigma-Aldrich, cat. no. A9731-10G)

#### Extracellular Matrix

- Matrigel (BD, cat. no. 356231)

#### Cell Detachment

- TrypLE express (Life Technologies, cat. no. 12604013)
- EDTA (Sigma, cat. no. E6635)
- Trypan blue (Sigma-Aldrich, cat. no. T8154-100ML)

#### Cytokines and Growth Factors

- GM-CSF (ImmunoTools, cat. no. 11343127)
- VEGF (ImmunoTools, cat. no. 11343667)
- SCF (ImmunoTools, cat. no. 11343327)
- BMP-4 (ImmunoTools, cat. no. 11345003)
- Interferon (IFN)-γ (R&D Systems, cat. no. 285-IF/CF)
- Tumor necrosis factor (TNF)-α (R&D Systems, cat. no. 210-TA/CF)
- IL-1β (R&D Systems, cat. no. 201-LB/CF)
- Prostaglandin E_2_ (TOCRIS, cat. no. 2296)
- IL-4 (Peprotech, cat. no. 200-04-500)
- IL-10 (ImmunoTools, cat. no. 11340105)
- Rapamycin (Sigma-Aldrich, cat. no. R0395)
- 1α,25-dihydroxyvitamin D_3_ (Sigma-Aldrich, cat. no. D1530)
- Dexamethasone (Sigma-Aldrich, cat. no. D4902)

#### Antibodies and Microbeads

- CD11c biotin (Miltenyi Biotec, cat. no. 130-092-413)
- Anti-biotin microbeads (Miltenyi Biotec, cat. no. 130-090-485)
- CD1c (BDCA-1)^+^ Dendritic Cell Isolation Kit (Miltenyi Biotec, cat. no. 130-090-506)

### Equipment

- Inverted phase-contrast microscope
- Laminar flow hood with HEPA filter for sterile cell culture work
- Standard cell culture incubator (37°C, 5% CO_2_, ≥95% humidity)
- Bench-top centrifuge with capacity for 15 ml tubes
- Flow cytometer (FACS Caliber, BD Biosciences)
- Magnetic separator, e.g., QuadroMACS (Miltenyi Biotec, cat. no. 130-090-976)
- MS columns (Miltenyi Biotec, cat. no. 130-042-401)
- Water bath (37°C)
- Hemocytometer
- Coverglass slips 12 mm (Fisher, cat. no. 12-545-82)
- Cell Bind 6-well plates (Corning, cat. no. CLS3335)
- Ultra-low attachment 6 well plates (Corning, cat. no. CLS347)
- Cell scraper (Corning, cat. no. CLS3010)
- Tubes, 15 and 50 ml (BD Falcon, cat. nos. 352095 and 352070)
- Plastic disposable pipettes 5, 10, and 25 ml (Corning, cat. no’s. 4487, 4488, 4489)
- Disposable sterile 0.22 µm filtration systems for volumes of 150 and 500 ml (Corning, cat. nos. CLS430626 and CLS430521)
- Syringe filter 0.22 µm (Millipore, cat. no. SLGP033RS)
- Cell strainer 70 µm (BD, cat. no. 352350)

## Set-Up

### Materials

#### Differentiation Medium

Differentiation medium is composed of XVIVO-15 supplemented as outlined in Table 1. Since human iPSCs may be adversely affected by the routine use of antibiotics, their addition to the medium should be avoided if possible. Consequently, it is essential to rigorously maintain sterile technique when culturing and passaging iPSCs and to filter sterilize medium after the addition of individual components.

**Table 1:** Composition of differentiation medium.

Component	Volume (ml)	Final concentration
XVIVO-15	1,000	
Sodium pyruvate	10	1 mM
Non-essential amino acids	10	0.1 mM
l-glutamine	10	2 mM
2-Mercaptoethanol	1	0.05 mM

#### Matrigel Stock

Thaw a 10 ml vial of matrigel on ice (this may take up to 4 h). Once thawed, add an equal volume of ice-cold Knockout DMEM to the matrigel solution using a pipette that has been kept at 4°C. Aliquot 1 ml into 1.8 ml Eppendorf tubes, placed on ice. Store at −80°C.

IMPORTANT! Note that matrigel rapidly solidifies above 4°C and must, therefore, be kept on ice at all times. To minimize solidification while aliquoting, all tips, pipettes, vials, and racks should be cooled to 4°C prior to use. During the aliquoting procedure, vials must be kept on ice and transferred to −80°C for storage as soon as possible.

#### 0.02% EDTA Solution (wt/vol)

Dissolve 1 g of EDTA in 500 ml of PBS to make 0.2% solution. Add 6N NaOH dropwise, while stirring until EDTA has dissolved. If necessary, adjust pH to 7.0 using 1M HCl. Autoclave to sterilize. Prepare a 1:10 dilution in PBS to make 0.02% solution of EDTA.

#### Y-27632 [Rho-Associated Kinase (ROCK) Inhibitor]

Dissolve 1 mg of Y-27632 in 314 µl of PBS (pH 7.2) to prepare a 10 mM stock solution. Store at −80°C for up to 3 months. This solution should be diluted 1:1,000 to yield a final concentration of 10 nM.

#### Granulocyte Macrophage Colony-Stimulating Factor (GM-CSF)

Dissolve 50 µg of lyophilized rhGM-CSF in 1 ml sterile PBS + 0.1% HSA to produce a stock solution of 50 µg/ml. Aliquot into 50 µl and 100 µl aliquots and store at −80°C. Avoid freeze-thaw cycles.

#### Vascular Endothelial Growth Factor (VEGF)

Dissolve 50 µg of lyophilized rhVEGF in 1 ml of sterile PBS + 0.1% HSA to produce a stock of 50 µg/ml. Aliquot into 50 and 100 µl aliquots and store at −80°C. Avoid freeze-thaw cycles.

#### Stem Cell Factor (SCF)

Dissolve 50 µg lyophilized rhSCF in 1 ml sterile PBS + 0.1% HSA to produce a stock solution of 50 µg/ml. Aliquot into 50 and 100 µl aliquots and store at −80°C. Avoid freeze-thaw cycles.

#### Bone Morphogenetic Protein 4 (BMP-4)

Dissolve 50 µg of lyophilized rhBMP-4 in 500 µl sterile PBS + 0.1% HSA to produce a stock solution of 100 µg/ml. Aliquot into 50 and 100 µl aliquots and store at −80°C. Avoid freeze-thaw cycles.

#### Prostaglandin E_2_ (PGE_2_)

Dissolve lyophilized PGE_2_ in DMSO to produce a stock solution of 5 mg/ml. Aliquot into 100 µl aliquots to serve as a 10× stock solution and store at −80°C. When required, thaw a single aliquot and dilute in 900 µl of PBS + 0.1% HSA to produce a working stock of 500 µg/ml.

IMPORTANT! DMSO is toxic and can penetrate the skin. Direct contact should, therefore, be avoided by wearing appropriate gloves.

#### IL-1β

Dissolve 50 µg rhIL-1β in 1 ml sterile PBS + 0.1% HSA to produce a stock of 50 µg/ml. Aliquot into 50 and 100 µl aliquots and store at −80°C. Avoid freeze-thaw cycles.

#### Interferon-γ

Dissolve 1 mg IFN-γ in 2.8 ml sterile PBS + 0.1% HSA and dilute solution to 10 ml with PBS + 0.1% HSA to produce a stock solution of 100 µg/ml. Aliquot into 50 and 100 µl aliquots and store at −80°C. Avoid freeze-thaw cycles.

#### Tumor Necrosis Factor-α

Dissolve the lyophilized rhTNF-α in sterile PBS + 0.1% HSA to produce a stock solution of 100 µg/ml. Aliquot into 50 and 100 µl aliquots and store at −80°C. Avoid freeze-thaw cycles.

#### Interleukin-4

Dissolve 500 µg of lyophilized rhIL-4 in 500 µl sterile PBS + 0.1% HSA. Dilute to 5 ml with PBS + 0.1% HSA to produce a stock of 100 µg/ml. Aliquot into 50 and 100 µl aliquots and store at −80°C. Avoid freeze-thaw cycles.

#### Rapamycin

Dissolve in 99% ethanol to produce a stock of 1 mg/ml and distribute into 100 µl aliquots. Store at −20°C and avoid freeze-thaw cycles. For further use after thawing, dilute a 100 µl vial with 900 µl of differentiation medium to produce a working stock that should be further diluted 1:1,000 in cultures to yield a final concentration of 100 ng/ml.

#### 1α,25-Dihydroxyvitamin D_3_ (VD_3_)

Dissolve in 99% ethanol to produce a 100 µM stock. Distribute into 10 µl aliquots and store at −20°C. Avoid freeze-thaw cycles.

#### Dexamethasone

Dissolve in 99% ethanol to produce a 10 mM stock. Distribute into 50 µl aliquots and store at −80°C avoiding freeze-thaw cycles.

#### Interleukin-10

Dissolve in sterile PBS + 0.1% HSA to produce a stock solution of 50 µg/ml. Aliquot into 50 and 100 µl aliquots and store at −80°C. Avoid freeze-thaw cycles.

#### 2-Mercaptoethanol

Dilute 70 µl of 2-ME in 20 ml of PBS to make a 1,000× stock solution.

IMPORTANT! 2-ME is highly toxic. Avoid inhalation and all contact with skin. Always use a fume hood to prepare a stock solution.

#### Rinsing Buffer

Dilute stock EDTA in PBS to yield a final concentration of 0.02% (w/v). Keep buffer refrigerated at 4°C and place on ice while in use.

#### Column Buffer

Prepare a 0.5% (w/v) solution of BSA in 0.02% (w/v) EDTA by carefully sprinkling the powder onto the surface and allowing it to dissolve slowly over time. Keep buffer refrigerated at 4°C and place on ice while in use.

### Equipment

#### Preparation of Matrigel-Coated 6-Well Plates

For the routine passage of human iPSCs, 6-well tissue culture plates may be coated with matrigel prior to use as follows and stored at 4°C for up to 2 weeks:

(1) Place a frozen vial of matrigel stock (stored at −80°C) on ice and allow to thaw, while never allowing the temperature to rise above 4°C. This process may take up to 3 h.
(2) Once thawed, transfer the contents of the vial, using a cold 1,000 µl pipette tip, into a pre-cooled 50 ml falcon tube containing 10 ml Knockout DMEM at 4°C. Make up the volume to 30 ml using Knockout DMEM to give a final dilution of 1:30. Mix well using a cold 10 ml pipette.
(3) Aliquot 1 ml of diluted matrigel into each well of a 6-well cell-bind plate. Tap the plate gently to ensure even dispersal of the matrigel over the entire surface of each well and add a further 1 ml of Knockout DMEM.
(4) A single vial of matrigel may be used to coat five 6-well plates. The plates need to be incubated at room temperature for at least 1 h. Plates can be sealed using cling film to prevent evaporation and stored at 4°C until required.
(5) Taking care not to allow the surface of wells to dry out, aspirate the matrigel solution from the plate immediately before use and replace with culture medium.

IMPORTANT! Since matrigel solidifies above 4°C, it must be kept on ice at all times. To minimize solidification during the coating procedure, all tips, pipettes, vials, and racks used must be cooled to 4°C prior to use. Knockout DMEM must also be kept at 4°C. Take care to avoid creating bubbles, as these can result in uneven coating of the wells.

## Detailed Protocol of the Procedure

### (A) Expansion of human iPSCs (6–7 days)

(1) Thaw a vial of human iPSCs onto the wells of a matrigel-coated 6-well cell bind plate in 3 ml of mTeSR1 medium per well, as described previously (35). It may take some days for colonies to become visible, but once established they should have a flat appearance, the cells toward the center becoming so closely packed that their boarders are difficult to discern (Figure 1B, top left). Culture the cells in mTeSR1 medium until colonies are of substantial size, but have yet to touch one another. Feed established iPSCs every day by removing 1 ml of spent medium from the well and adding 1 ml of fresh mTeSR1.
(2) Although iPSC lines may differ subtly in their growth characteristics, in our hands, most cell lines require passaging every 6–7 days at a 1:12 dilution, using 0.02% EDTA in PBS to dissociate the colonies.
  (i) Add Y-27632 to mTesR1 to produce a final concentration of 10 µM. Filter sterilize using a 0.22 µm syringe filter.
    IMPORTANT! Human iPSCs are especially sensitive to dissociation into a single cell suspension. The ROCK inhibitor Y-27632 has been shown to protect cells from dissociation-induced apoptosis (36) and is, therefore, routinely added during passaging. Y-27632 may also be added to medium upon thawing of the iPSC line in order to enhance viability but should be removed once the cells have adhered to matrigel.
  (ii) Aspirate mTesR1 from wells containing iPSC colonies and rinse with PBS. Add 1 ml of 0.02% EDTA to the well and leave for 30 s before removing by aspiration and rinsing again with PBS.
  (iii) Add 1 ml of mTesR1 containing 10 µM Y-27632 to each well. Scrape the colonies from the surface to form clusters using a sterile cell scraper.
    IMPORTANT! Take care not to be too harsh with mechanical scraping which can cause significant cell death. Make sure to release the colonies but ensure that they are not reduced to a single cell suspension.
  (iv) Gently transfer the cell clusters suspended in mTesR1 into a 50 ml falcon tube using a 10 ml pipette. Wash the well with 1 ml of mTesR1 containing Y-27632 to collect any remaining clusters of cells. Cell clusters from the same passage can be pooled together from multiple wells.
  (v) Top up the tube containing the clusters with an appropriate volume of mTesR1 containing Y-27632 to achieve a 1:12 dilution of the original cell suspension.
  (vi) Pipette the suspension gently to ensure that clusters do not settle to the bottom of the tube and dispense 2 ml into each well of a fresh matrigel-coated 6-well plate, gently agitating the plate to ensure even distribution of the clusters.
  (vii) Incubate the plate in a humidified incubator at 37°C, 5% CO_2_.
    IMPORTANT! The iPSCs should be expanded until the number of wells required for differentiation is achieved. As a rough estimate, three wells of iPSCs in a 6-well plate provide sufficient material to establish a single well of embryoid bodies in a 6-well ULA plate.

### (B) Set-up of cultures for the differentiation of DCs (45 min)

All reagents used to establish differentiation cultures should be maintained at room temperature.

(1) To estimate total number of iPSCs in culture, sacrifice a single well for counting purposes by dissociating colonies of iPSCs into a single cell suspension. The cells from this well should not be included in the differentiation culture as they have a greater propensity for apoptosis.
  (i) Aspirate the culture medium from a single well of iPSCs. Wash the well with 1 ml of PBS. Remove the PBS and add 1 ml of TrypLE express. Incubate the plate at 37°C for 5 min or until dissociated into a single cell suspension.
  (ii) Add 1 ml of Knockout DMEM to the well and fully dissociate the cells by pipetting up and down with a Gilson pipette and a 1,000 µl pipette tip.
  (iii) Transfer the cells to a 1.8 ml Eppendorf tube. Mix 20 µl of cell suspension with 20 µl of trypan blue and count the number of cells using a standard hemocytometer. Calculate the total number of cells in one well.
(2) Use the resulting cell counts to estimate the total number of cells available for differentiation. Working on the assumption that 3 × 10^6^ cells should be seeded per well for the purposes of differentiation, calculate the total number of wells that can be established.
(3) Prepare sufficient mTesR1 to allow for 4 ml per well of differentiation cultures. Supplement the mTesR1 with 50 ng/ml of rhGM-CSF, 50 ng/ml rhVEGF, 50 ng/ml rhBMP-4, and 20 ng/ml rhSCF and filter sterilize using a 0.22 µm filter. Additionally, 10 µM Y-27632 may be added to the medium to minimize dissociation-induced apoptosis during the early stages of differentiation.
  IMPORTANT! Differentiation cultures are initially established in mTesR1 medium, to which the cells have become accustomed during routine culture. Setting up the cultures in XVIVO-15 differentiation medium causes substantial cell death and may lead to failure of the differentiation process. XVIVO-15 is, therefore, introduced gradually by using it to replace mTeSR1 during routine feeding of the cultures, a process which appears to be better tolerated by iPSCs.
(4) Harvest iPSC colonies as described in A step 2 (ii) and add 1 ml of growth factor supplemented mTeSR1. Carefully scrape colonies from the surface to form clusters.
(5) Pool clusters of iPSCs from multiple wells into a 50 ml falcon tube and top up with the growth factor-supplemented mTesR1 medium to give the final volume required. The clusters should be visible to the naked eye at this stage. Using a 10 ml pipette and pipette controller set on low speed, dispense 4 ml of this mixture into the appropriate number of wells of 6-well ULA plates. Gently pipette the cell suspension up and down between plates to ensure the even distribution of clusters.
(6) Place the plates in an incubator and gently rock by hand back and forth and from side to side to ensure even dispersal of clusters across each well. Cultures should be incubated at 37°C, 5% CO_2_ in a humidified environment.
  IMPORTANT! Uneven dispersal of colonies may result in the clumping and adherence of cell clusters, potentially hampering the differentiation process.

### (C) Maintenance of differentiation cultures (30 min every 2 days)

Differentiation cultures should be fed regularly every 2–3 days from day 2 of culture until they are harvested around days 21–24. However, if the culture medium consistently shows signs of exhaustion, the frequency of feeding should be increased. Toward the end of the differentiation, cultures are fed every 2 days. The growth factors in the differentiation medium are removed progressively until only GM-CSF remains, causing the concentration of each growth factor to decrease through the course of differentiation, according to a pre-defined schedule (Figure 1A).

(1) For the first feed, prepare sufficient medium by supplementing the appropriate volume of XVIVO-15 differentiation medium with rhBMP-4, rhVEGF, rhSCF, and rhGM-CSF at the concentrations outlined in B step 3. Each well requires the addition of 2 ml of fresh differentiation medium. Filter sterilize the medium through a 0.22 µm filter and warm it in a 37°C water bath prior to use.
(2) For the first feed (typically 2 days after establishing the cultures), carefully add 2 ml of differentiation medium per well to the medium used to set up the culture.
(3) On subsequent occasions (days 4–24), remove 2 ml of differentiation medium from each well. Carefully, aspirate the medium from the surface with a 10 ml pipette, taking care not to remove any cell clusters or embryoid bodies that may have developed (Figure 1B, top center). Add 2 ml of fresh differentiation medium to each well containing the full combination of growth factors.
(4) Feed cultures every 2–3 days, as required. The amount of medium withdrawn and replaced can be increased to 3 ml if the medium shows signs of exhaustion. From day 5 onward, remove rhBMP-4 from the differentiation medium. Recombinant human VEGF and rhSCF are successively removed from days 9 and 14 of culture, respectively.
  IMPORTANT! Cultures may contain a significant amount of cell debris during the early stages of differentiation, which is entirely normal.
(5) Around days 10–14 of culture, the appearance of small, round cells of hematopoietic origin should be observed. From days 14 to 18, macrophages with characteristic “fried egg” morphology and firm adherence to the tissue culture plate may start to appear. Numbers of macrophages may vary significantly between differentiations, even when using the same iPSC line. At the point of their appearance, add rhIL-4 to the differentiation cultures. IL-4 is introduced gradually, starting at 25 ng/ml which, in subsequent feeds, can be increased to 50, 75, and finally, 100 ng/ml as the number of DC precursors and immature DCs begins to increase.

### (D) Harvesting DC precursors and immature DCs (25 min)

(1) By days 21–24, significant numbers of DC precursors and immature DCs should be visible in the wells, frequently forming a “halo” surrounding individual embryoid bodies (Figure 1B, top right). Adherent macrophages may also be visible, although the addition of rhIL-4 appears to limit their numbers while further promoting the differentiation of DCs.
  IMPORTANT! The timing of events may vary significantly between experiments and even between wells cultured in parallel as part of the same experiment. Although we routinely harvest cultures around day 24, it is not uncommon to wait until day 30 for sufficient DC precursors and immature DCs to be available for harvesting.
(2) Harvest DC precursors and immature DCs by gently pipetting cultures up and down using a 10 ml pipette and pipette controller set on low speed, to remove non-adherent and weakly adherent DCs while leaving macrophages firmly adherent. Pass the cells through a 70 µm cell strainer to remove large debris and embryoid bodies.
(3) Centrifuge the cell suspension at 300 *g* for 5 min at 4°C and discard the supernatant. Resuspend the cells in 10 ml of fresh XVIVO-15 differentiation medium containing 50 ng/ml rhGM-CSF and 100 ng/ml rhIL-4.
(4) Estimate cell numbers by using trypan blue exclusion following the procedure outlined in section B, step 1 (iii). Add differentiation medium to produce a final cell number of 2.5 × 10^5^ cells per milliliter of medium.
(5) Plate 4 ml of cell suspension into each well of 6-well cell-bind plates, such that each well contains 1–2 × 10^6^ cells (Figure 1B, bottom left). Incubate cells at 37°C, 5% CO_2_ in a humidified incubator.
  IMPORTANT! Cell-bind plates are used at this stage to encourage any macrophages that may have been carried over during harvesting to adhere.

### (E) Pharmacological modulation and maturation of ipDCs (7 days)

(1) Immature DCs harvested between days 21 and 30 of culture may be functionally modulated using pharmacological agents to reinforce a tolerogenic phenotype. Agents including rhIL-10, rapamycin, dexamethasone, and VD_3_ may be added to cultures following the plating of immature ipDC onto cell-bind plates (Table 2).
  (i) For modulation with VD_3_, add 4 µl of 100 µM VD_3_ stock to each well containing 4 ml of medium to produce a final concentration of 100 nM. VD_3_ is added on days 0 and 3 following the plating of DCs onto cell-bind plates.
  (ii) For modulation with dexamethasone, add 40 µl of 10 mM dexamethasone stock to each well containing 4 ml of medium to produce a final concentration of 100 µM. Dexamethasone is added on day 3 of culture following the transfer of DCs to cell-bind plates.
  (iii) For modulation with rapamycin, dilute the 1 mg/ml stock solution 1:1,000 in medium, of which 40 µl are added to each well in order to produce a final concentration of 10 ng/ml. Rapamycin is added on day 3 following the harvesting of DCs onto cell-bind plates.
  (iv) For modulation with rhIL-10, add 16 µl of stock cytokine at 50 ng/ml to each well containing 4 ml of medium to produce a final concentration of 200 pg/ml. IL-10 is added on day 3 of culture, following the plating of DCs onto cell-bind plates.
(2) DCs can be matured with or without prior pharmacological treatment, by culturing for 48 h in a cocktail of inflammatory cytokines consisting of 50 ng/ml rhTNF-α, 1 µg/ml PGE_2_, 10 ng/ml rhIL-1β, and 20 ng/ml rhIFN-γ.
  (i) Determine the number of wells to be matured and for each well transfer 0.5 ml of XVIVO-15 differentiation medium containing 50 ng/ml rhGM-CSF and 100 ng/ml rhIL-4 to a 15 ml falcon tube.
  (ii) Add rhTNF-α, PGE_2_, rhIL-1β, and rhIFN-γ to the medium to produce a stock 9 times the final concentration required. Filter sterilize using a 0.22 µm syringe filter.
  (iii) Add 0.5 ml of stock cytokine cocktail to each well requiring maturation to yield the desired final concentration of cytokines.
    IMPORTANT! Do not remove any medium from the wells.
(3) After 48 h, harvest immature and mature DCs by gently pipetting cultures up and down using a 10 ml pipette with the pipette controller set to low speed, so as to remove non-adherent and weakly adherent DCs, while leaving behind firmly adherent macrophages.

**Table 2:** Pharmacological agents used for modulating dendritic cell function, showing their stock concentrations, final concentrations in culture, and the days on which they are added.

Agent	Stock conc.	Final conc.	Day(s) added (post-harvest)
Rapamycin	1 mg/ml	100 ng/ml	3
VD_3_	100 µM	100 nM	0 and 3
Dexamethasone	10 mM	100 µM	3
Interleukin-10	50 µg/ml	200 pg/ml	3

### (F) Purification of CD11c^+^ ipDCs (1.5 h)

(1) Estimate the total number of ipDCs obtained after harvesting using trypan blue exclusion according to B step 1 (iii).
(2) Label cells with biotinylated CD11c antibody following the manufacturer’s instructions:
  (i) Pass the cells through a 70 µm cell filter to remove debris and clusters of cells.
  (ii) Centrifuge DCs at 300 *g* for 10 min and carefully aspirate and discard the supernatant.
  (iii) Resuspend 10^7^ cells in 100 µl of column buffer and transfer them to a sterile Eppendorf tube.
  (iv) Add 10 µl of biotinylated CD11c monoclonal antibody to 100 µl of cell suspension. Mix gently by pipetting up and down several times and incubate at 4°C for 10 min, either by placing on ice or in a refrigerator. If cell yields exceed 10^7^ cells, scale up the volumes of buffer and antibody accordingly.
    IMPORTANT! Work quickly and keep the cells cold to prevent capping and shedding of bound antibody.
  (v) Add 1 ml of cold column buffer to the tube to wash the cells. Centrifuge the cell suspension at 300 *g* for 10 min and discard the supernatant. Repeat this step 2 further times to remove unbound antibody.
(3) Incubate the cells with anti-biotin microbeads and purify the cells using magnetic bead-based separation:
  (i) Resuspend the cell pellet in 80 µl of column buffer and add 20 µl of anti-biotin microbeads to 10^7^ cells. If working with more cells, scale up the volumes of buffer and microbeads accordingly.
  (ii) Mix the cell suspension and microbeads by gently pipetting up and down several times and incubate at 4°C for 15 min, either by placing on ice or in a refrigerator.
  (iii) Add 1 ml of cold column buffer to the tube to wash the cells. Centrifuge the cell suspension at 300 *g* for 10 min. Discard the supernatant and repeat this step 2 further times.
  (iv) Resuspend the cell pellet in 500 µl of cold column buffer.
  (v) Place a fresh MS column, with a maximum capacity of 10^7^ cells, in the magnetic field of a magnetic separator.
  (vi) Pass 500 µl of rinsing buffer through the column.
  (vii) Add the 500 µl of cell suspension to the column and collect flow-through in a 15ml falcon tube: this represents the unlabeled cell fraction.
  (viii) Wash the column by allowing 500 µl of rinsing buffer to flow through while it is still attached to the magnetic separator. Discard the eluent.
  (ix) Remove the column from the magnetic separator. Add 1 ml of rinsing buffer to the column and immediately flush out the microbead-labeled cells by gently depressing the plunger. Collect the eluent in a fresh 15 ml falcon tube. This represents the purified fraction of CD11c^+^ cells.
    IMPORTANT! It is advisable to assess the purity of the population, preferably using flow cytometric analysis. Fluorescently labeled streptavidin will displace microbeads from the surface of the cells since its affinity for biotin is orders of magnitude higher than that of the anti-biotin monoclonal antibody, thereby permitting the percentage of CD11c^+^ cells to be determined. Typically, a single round of purification yields a population enriched to ~90% purity (Figure 2C).

### (G) Purification of ipDCs by depletion of CD1c^+^ cells (1.5 h)

(1) After harvesting, estimate the total number of DCs obtained from a differentiation using trypan blue exclusion, as described in B step 1 (iii).
(2) Label the cells with CD1c monoclonal antibody supplied in the CD1c (BDCA-1)^+^ Dendritic Cell Isolation Kit:
  (i) Pass the cells through a 70 µm filter to remove cell clumps.
  (ii) Centrifuge DCs at 300 *g* for 10 min. Carefully aspirate and discard the supernatant.
  (iii) Resuspend the cells to a density of 10^7^ cells in 200 µl of cold column buffer and transfer to a 15 ml falcon tube.
  (iv) Add 10 µl of the FcR Blocking Reagent provided in the kit and 10 µl of CD1c-biotin to the cell suspension. Gently mix the cells and antibody by pipetting up and down several times and incubate at 4°C, either on ice or in a refrigerator for 15 min. If working with more cells, scale up volumes of buffer and antibody accordingly.
    IMPORTANT! Work quickly, keeping the cells cold and using pre-cooled solutions to prevent capping and shedding of bound antibody.
  (v) Add 4 ml of cold column buffer to the tube to wash the cells. Centrifuge the cell suspension at 300 *g* for 10 min and discard the supernatant. Repeat this step 2 further times.
(3) Incubate the cells with anti-biotin microbeads and remove the labeled cells using magnetic bead-based separation:
  (i) Resuspend cell pellet in 400 µl of column buffer.
  (ii) Add 10 µl of anti-biotin microbeads to a maximum of 10^7^ cells. If working with more cells, scale up the volumes of buffer and antibody accordingly.
  (iii) Mix the cell suspension and microbeads by aspirating up and down gently a couple of times and incubate at 4°C for 15 min, either on ice or in a refrigerator.
  (iv) Add 4 ml of cold column buffer to the tube to wash the cells. Centrifuge the cell suspension at 300 *g* for 10 min and discard the supernatant. Repeat this step 2 further times.
  (v) Resuspend cell pellet in 500 µl of cold column buffer.
  (vi) Prepare the MS column as described in F steps 3 (v)–(vi).
  (vii) Add the cell suspension to the column and collect the flow-through in a 15-ml falcon tube.
  (viii) Wash the column by adding 500 µl of rinsing buffer to the column, while it is still attached to the magnetic separator. Collect the eluent in a fresh 15 ml falcon tube and combine with the eluent from step (vii). This represents the unlabeled cell fraction that contains the CD141^+^ cells.
  (ix) Remove the column from the magnetic separator and flush out the microbead-labeled cells by gently depressing the plunger. This fraction contains the CD1c^+^ subset.
    IMPORTANT! It is advisable to assess the purity of either population before use, preferably by flow cytometry. A single round of negative selection typically enriches the CD141^+^ subset to >70% purity (Figure 2D). Although this may be further improved by additional rounds of separation, such purity is generally at the expense of cell yields, which may decrease substantially. It is essential, therefore, to determine the cell numbers and level of purity required for each application and plan experiments accordingly.

## Timing

Timings will vary depending on the magnitude of the differentiation culture and are, therefore, expressed as the time required for the handling of a 6 well plate.

(A) Expansion of human iPSCs: 50–60 min/6–7 days
  Step 1: 20 min
  Step 2: 30–40 min
(B) Establishment of differentiation cultures: ~45 min
  Step 1: 15 min
  Steps 2–6: 20–30 min
(C) Maintenance of differentiation cultures: ~50 min every 2 days/~22 days
  Steps 1–2: 10–20 min
  Steps 3–5: 30 min every 2 days
(D) Harvesting of DC precursors and immature DCs: 25 min
  Steps 1–3: 15 min
  Steps 4–5: 10 min
(E) Pharmacological modulation and maturation of ipDCs: 30 min/7 days
  Step 1: 5 min
  Step 2: 10 min
  Step 3: 5–10 min
(F) Purification of CD11c^+^ ipDCs by positive selection: 1.5 h
  Step 1: 10 min
  Step 2: 35 min
  Step 3: 45 min
(G) Purification of CD141^+^ ipDCs by negative selection of CD1c^+^ cells: 1.5 h
  Step 1: 10 min
  Step 2: 35 min
  Step 3: 45 min

## Expected Results

**Figure 3:**
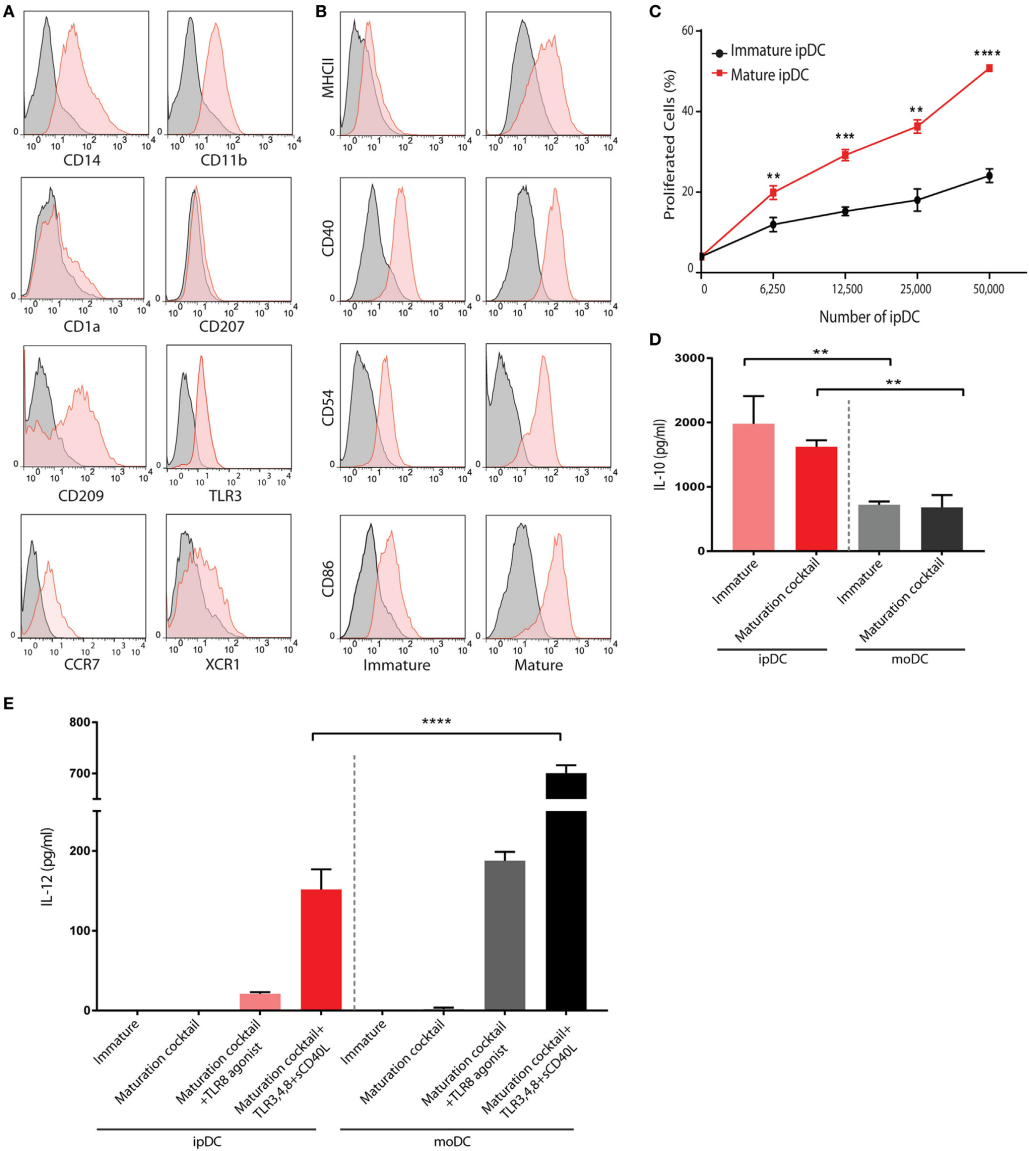
Phenotypic and functional characterization of human iPSC-derived DC (ipDCs). **(A)** Histograms depicting the expression of lineage markers by ipDCs gated on the CD11c^+^ population. Levels of expression of individual markers are shown as red histograms, while non-specific staining by isotype controls is shown as gray histograms. **(B)** Maturation of ipDCs in response to a cocktail of inflammatory cytokines showing upregulation of MHC class II, CD40, CD54, and CD86 compared to immature cells. Red histograms represent levels of expression of markers by CD11c-gated ipDCs, while isotype controls are shown in black. All FACS plots are representative of three independent experiments. **(C)** ipDCs display increased immunostimulatory capacity upon maturation as evidenced by enhanced proliferation of naïve CD4^+^ T cells co-cultured in triplicate with mature (red line) or immature cells (black line). Data are representative of three independent experiments. Statistical significance was determined using the Mann–Whitney *U* test. **(D)** Interleukin (IL)-10 secretion by ipDCs in response to maturation stimuli. Immature ipDCs were cultured in triplicate with or without a cocktail of pro-inflammatory cytokines consisting of PGE_2_, tumor necrosis factor (TNF)-α, IL-1β, and interferon (IFN)-γ. Equivalent numbers of monocyte-derived DCs (moDCs) were cultured in parallel for 18 h and levels of IL-10 quantified from culture supernatants by standard ELISA. Plots are representative of at least three independent experiments. Statistical analysis was performed using parametric *T* tests with Welch’s correction (**p < 0.01). **(E)** Secretion of IL-12p70 in response to immunological challenges. Immature ipDCs were cultured in triplicate either alone, with a cocktail of pro-inflammatory cytokines or with maturation cocktail further supplemented with toll-like receptor (TLR) agonists and soluble CD40L. Controls consisted of equivalent numbers of moDCs cultured in parallel. Levels of IL-12p70 were quantified from culture supernatants by standard ELISA. Data are representative of three independent experiments, and statistical analysis was performed using parametric *T* tests with Welch’s correction. (*p < 0.05; **p < 0.01; ***p < 0.001; ****p < 0.0001).

The application of our protocols to human iPSCs typically yields DCs displaying some of the features of the CD141^+^ subset within approximately 24 days of culture, that may be enriched through negative selection of CD1c^+^ cells to yield an “untouched” population, unaffected by cross-linking of surface CD141 (Figure 2D). Flow cytometry reveals that, in addition to CD141, these cells constitutively express TLR3 and the chemokine receptor XCR1 (Figure 3A) as reported previously (32), suggesting that they are analogous to the subset of DCs endowed with cross-presentation capacity (14–17, 32). Interestingly, these cells express barely detectable levels of CD1a and CD207, distinguishing them from dermal DCs and Langerhans cells, respectively, but consistently express both CD14 and CD209 (Figure 3A), recently found to be co-expressed by some populations of dermal DCs (37) and to define “regulatory” DCs in the skin (23), an indication that CD141^+^ ipDC may fail to perfectly recapitulate all properties of the conventional CD141^+^ subset *in vivo*. Consistent with their capacity for antigen presentation, CD141^+^ ipDCs constitutively express the co-stimulatory molecules CD40, CD54, and CD86, which are upregulated upon exposure to a cocktail of inflammatory cytokines (Figure 3B). MHC class II is likewise upregulated upon maturation, provoking the activation and proliferation of naïve, CFSE-labeled allogeneic T cells in the mixed leukocyte reaction (MLR) (Figure 3C). Similar to CD141^+^ DCs in the skin (23), ipDCs constitutively secrete levels of IL-10 which are substantially higher than those produced by control populations of moDCs cultured in parallel (Figure 3D). Importantly, IL-10 has been shown to interfere with the initiation of Th1 responses (38) and to favor the polarization of naïve T cells toward a Treg phenotype (39, 40). Furthermore, upon maturation in response to inflammatory cytokines, ipDCs fail to secrete IL-12 required for Th1 polarization and CTL activation (41, 42), indeed, only maximal stimulation with a combination of inflammatory cytokines, TLR agonists and CD40 cross-linking is capable of eliciting significant IL-12 secretion (Figure 3E), reminiscent of reports of interstitial DCs including the CD141^+^ subset (18, 21). Importantly, the equivalent treatment of moDCs in parallel cultures consistently yields substantially higher levels of the pro-inflammatory cytokine (Figure 3E).

**Figure 4:**
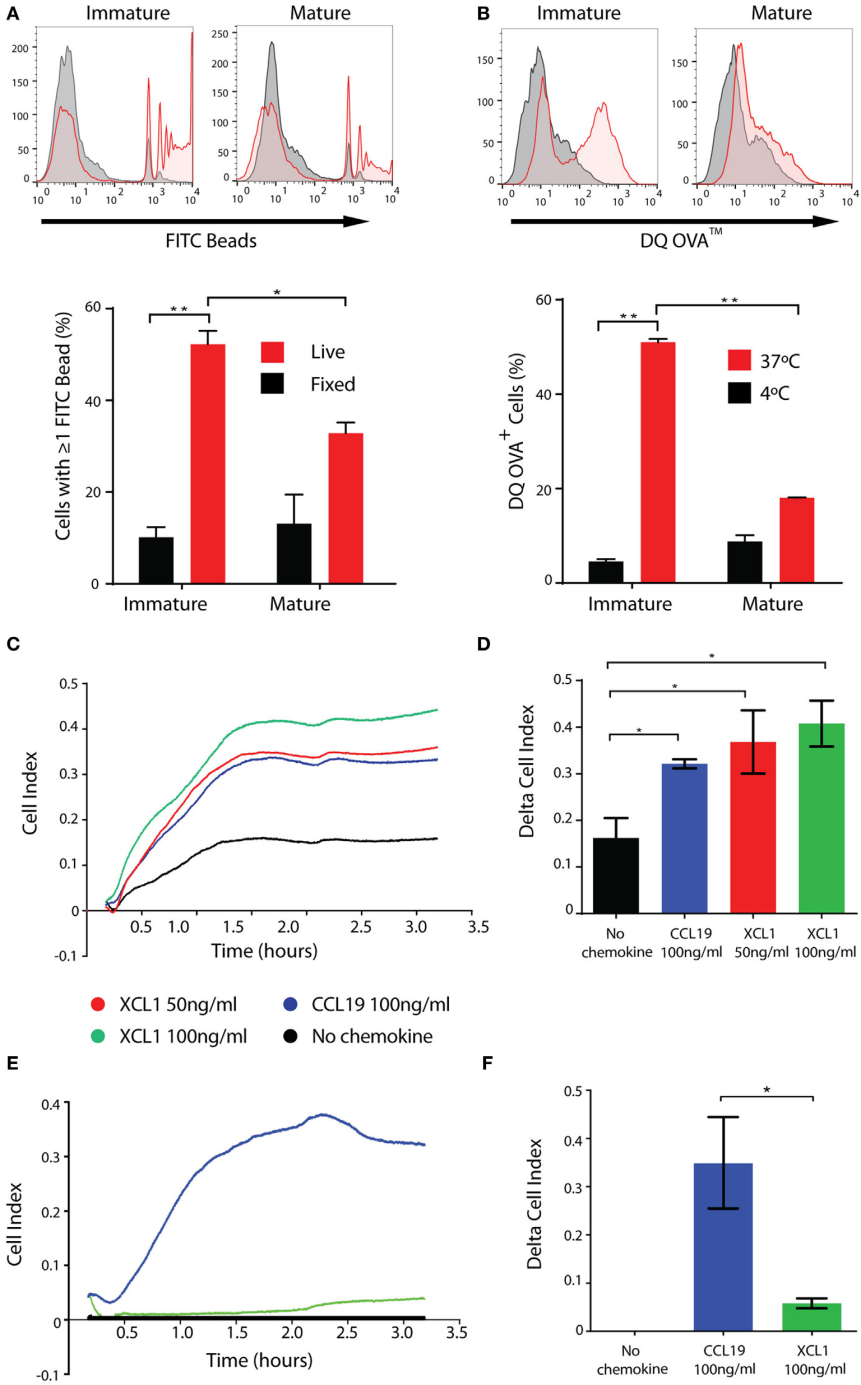
iPSC-derived DCs (ipDCs) show marked phagocytic and endocytic capacity and migrate in response to physiological stimuli. **(A)**. Representative histograms and bar chart showing phagocytosis of 2 µm diameter fluorescently labeled beads by CD11c^+^ ipDCs over a 3 h incubation period (red histograms and bars). Non-specific binding of beads was assessed using fixed ipDCs (grey histograms and black bars). Data are representative of three independent experiments consisting of triplicate cultures. Statistical analysis was performed using parametric *T* test with Welch’s correction. **(B)** Representative histograms and bar chart showing the endocytosis and proteolysis of DQ-OVA over a 30 min incubation period by CD11c^+^ ipDCs (red histograms and bars). Negative controls consisted of ipDCs incubated with DQ-OVA at 4°C (grey histograms and black bars). Data are representative of three independent experiments consisting of triplicate cultures. Statistical analysis was performed using parametric *T* test with Welch’s correction. **(C)** Chemotaxis of ipDCs in response to CCL19 and XCL1 over a 4 h period measured in real time using the xCELLigence Real-Time Cell Analyzer. **(D)** Comparison of delta cell index (max–min of cell index) for each chemokine compared to negative controls, incubated in the absence of added chemokines. **(E)** Chemotaxis of control monocyte-derived DCs (moDCs) cultured in parallel, in response to CCL19 and XCL1 measured in real time over a 4 h period. **(F)** Comparison of delta cell index for cultures of moDCs. All plots are representative of three independent experiments. Data represent the mean ± SD. Statistical analysis was performed using parametric *T* tests with Welch’s correction (*p ≤ 0.05; **p ≤ 0.001).

CD141^+^ DCs *in vivo* are characterized by their marked capacity for uptake and processing of both soluble and cellular antigens (19) and their chemotaxis in response to CCL19 and XCL1. Consistent with this remit, CD141^+^ ipDCs display significant capacity for the phagocytosis of fluorescently labeled latex beads which is abrogated upon fixation, more than 50% of immature cells being shown to phagocytose multiple beads over a 3 h incubation period, their propensity for phagocytosis decreasing following maturation, as previously reported (43, 44) (Figure 4A). DQ-OVA is a derivative of ovalbumin conjugated with boron-dipyrromethene, a photostable, pH insensitive dye which fluoresces following proteolytic cleavage (45) and therefore serves as a measure of antigen processing activity. Incubation of ipDCs expressing CD141 with DQ-OVA consistently reveals both significant uptake and processing of the substrate which is inhibited at 4°C and progressively lost upon maturation (Figure 4B).

In our hands, the migratory capacity of ipDCs is consistent with that reported previously for the CD141^+^ subset, as determined using electrical cell-substrate impedance sensing. The xCELLigence Real-Time Cell Analyzer measures electrical impedance caused by the migration of cells through pores 8 µm in diameter in a filter, in which is embedded a gold micro-electrode: the resulting arbitrary units of cell index provide a measure of the number of cells migrating across the filter in real time (46). Congruent with their expression of CCR7 (Figure 3A), ipDCs consistently migrate in response to a gradient of rhCCL19, known to guide DCs from interstitial tissues to the secondary lymphoid organs *in vivo* (47) (Figure 4C). Furthermore, ipDCs uniquely respond to the chemokine XCL1 in a dose-dependent manner (Figures 4C,D), confirming the functionality of surface XCR1. In contrast, moDCs cultured in parallel, respond reliably to CCL19 but do not migrate in response to XCL1 (Figures 4E,F), consistent with their failure to express the *XCR1* gene (16). *In vivo*, XCL1 is predominantly secreted by CD8^+^ T cells and acts as a chemo-attractant that is highly specific for CD141^+^ DCs, thereby enhancing the cross-presentation of antigen to the MHC class I-restricted T cell repertoire (48).

**Figure 5:**
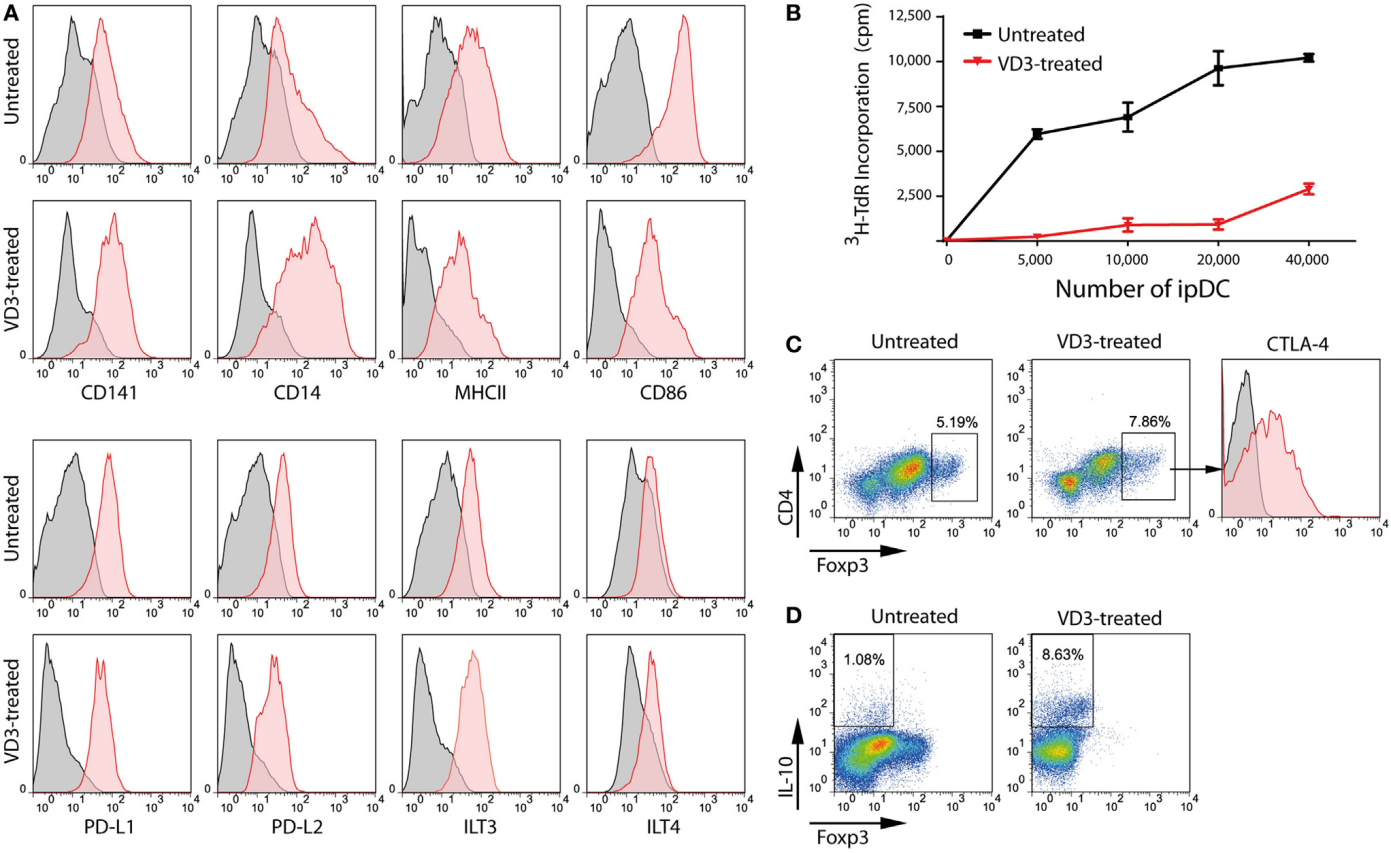
VD_3_ treatment of iPSC-derived DCs (ipDCs) reinforces a regulatory phenotype. **(A)** Representative histograms showing the impact of VD_3_ on expression of cell surface markers including co-stimulatory molecules and the inhibitory receptors PD-L1, PD-L2, immunoglobulin-like transcript (ILT)3, and ILT4. Levels of expression of individual markers are shown as red histograms, while non-specific staining by appropriately matched isotype controls is shown in grey. **(B)** Reduced immunostimulatory capacity of ipDCs following exposure to VD_3_ (red line) compared with untreated controls (black line), as determined by proliferation of naïve CD4^+^ T cells in the allogeneic mixed leukocyte reaction. **(C)** Polarization of naïve CD4^+^ T cells toward a regulatory T cell (Treg) phenotype following 5 days’ co-culture with either untreated or VD_3_-treated ipDCs followed by the addition of 75 ng/ml rhIL-2 for a further 2 days. Treg commitment was assessed by the upregulation of FoxP3 and surface CTLA-4. **(D)** Polarization of naïve CD4^+^ T cells toward a Tr1 phenotype, characterized by secretion of interleukin (IL)-10. Mature untreated and VD_3_-treated ipDCs were co-cultured with CD4^+^ T cells for 5 days followed by a 2-day treatment with 75 ng/ml of rhIL-2. On day 7, co-cultures were treated with 10 µg/ml of Brefeldin A, 700 ng/ml ionomycin, and 20 ng/ml phorbol 12-myristate 13-acetate for 5 h before being stained for intracellular IL-10.

Exposure to high levels of UV light promotes the local synthesis of VD_3_ within the skin which is known to be processed to its active form by resident DCs (49), potentially contributing to their regulatory function in the steady state. Accordingly, addition of VD_3_ to cultures of ipDCs, concomitant with their exposure to a maturation cocktail of pro-inflammatory cytokines, results in the further upregulation of CD14 (Figure 5A), the resulting CD14^hi^CD141^+^ phenotype having been identified previously as indicative of regulatory function (23). Furthermore, ipDCs exposed to VD_3_ during differentiation show resistance to maturation, as evidenced by the failure to upregulate MHC class II and costimulatory molecules, while showing marked expression of the inhibitory receptors programmed death ligand-1 (PD-L1), PD-L2, and immunoglobulin-like transcript (ILT)-3 (50–53) (Figure 5A). Since tolerance is, in essence, an *in vivo* phenomenon, the tolerogenicity of ipDCs can be determined unequivocally only from the outcome of future clinical trials. Nevertheless, *in vitro* correlates have been shown to have predictive value, especially in mouse models in which allograft rejection has been prevented by the administration of “regulatory” DCs differentiated from iPSCs (54, 55). In these studies, the administered DCs showed decreased capacity for effector T cell priming *in vitro* and polarization of responding T cells toward a Treg phenotype. Accordingly, ipDCs conditioned by exposure to VD_3_ consistently display reduced stimulatory capacity in the MLR compared to untreated controls (Figure 5B). Furthermore, in co-cultures with naïve peripheral blood T cells, VD_3_-treated ipDCs promote a modest increase in commitment of responding T cells toward a Treg phenotype, defined by co-expression of FoxP3 and CTLA-4 (Figure 5C), but elicite a substantial increase in Tr1 cells (56–58), as evidenced by the appearance of T cells stained positively for intracellular IL-10 (Figure 5D).

**Figure 6:**
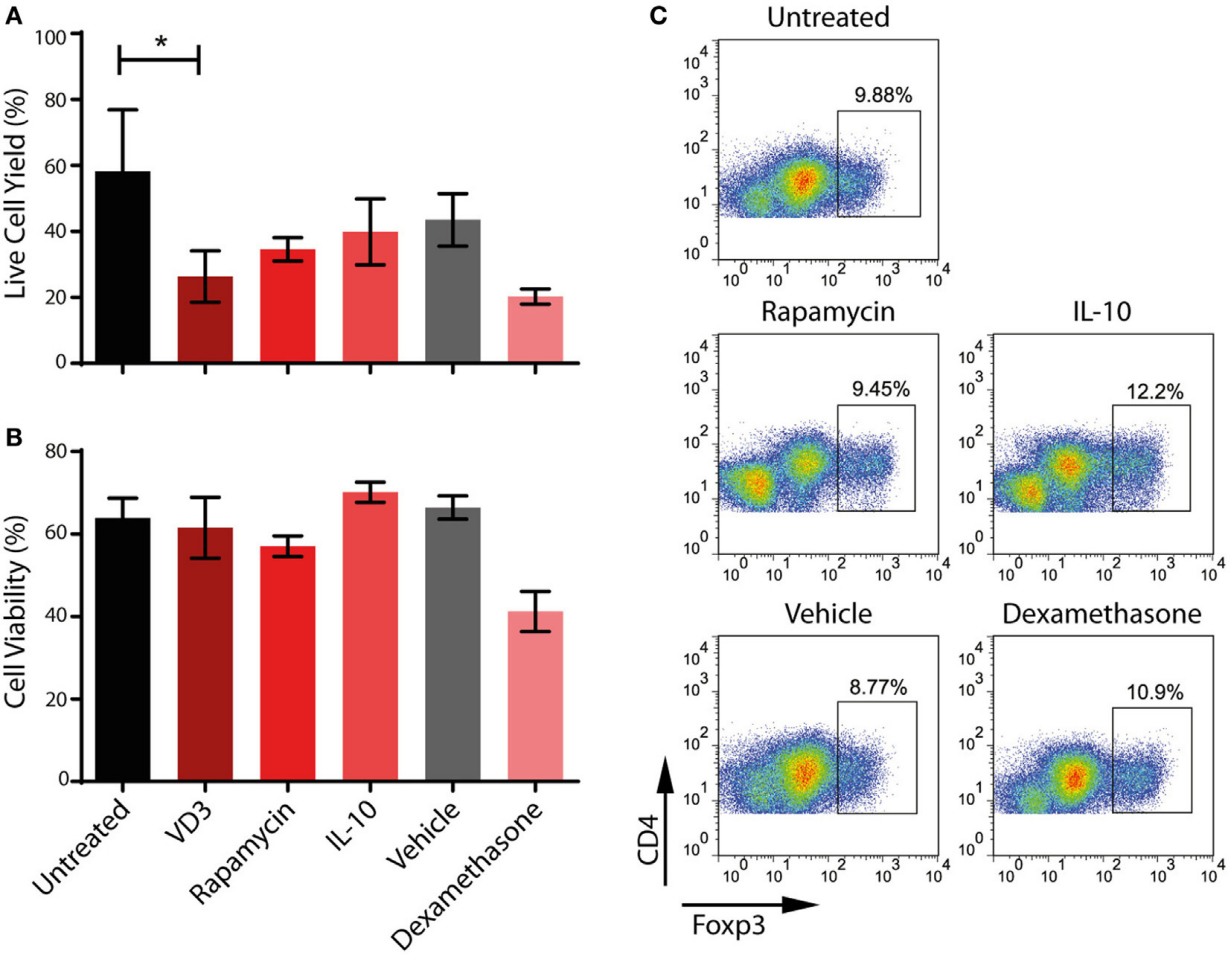
Impact on iPSC-derived DCs (ipDCs) of pharmacological agents known to favor a regulatory phenotype. **(A)** Yield of ipDCs expressed as a percentage of the number of precursors harvested from differentiation cultures, prior to exposure to pharmacological agents. **(B)** Viability of ipDCs following culture with pharmacological agents and subsequent maturation as determined by trypan blue exclusion. **(C)** Regulatory T cell (Treg) induction by ipDCs in co-cultures with naïve, allogeneic CD4^+^ T cells for 5 days followed by a 2-day treatment with 75 ng/ml of rhIL-2. Cells were harvested and stained for CD4 and intracellular Foxp3 and analyzed by flow cytometry. Data are representative of three independent experiments.

Although VD_3_ has been used successfully to modulate the activity of moDCs for use in clinical trials (59), we consistently find that its use alters the morphology of ipDCs while substantially increasing their adherence to plastic, even when using ULA plates. Furthermore, the yield of ipDCs is significantly reduced in the presence of VD_3_ compared to cultures differentiated in its absence (Figure 6A), a finding which has prompted us to explore the use of other pharmacological agents known to induce a tolerogenic phenotype (11). Treatment with dexamethasone compromises both the yield and viability of ipDCs (Figures 6A,B), while rapamycin has little discernible effect on their propensity for Treg induction (Figure 6C). In contrast, IL-10 is compatible with acceptable yields and viability, while modestly enhancing the polarization of naïve allogeneic T cells toward a Treg phenotype (Figure 6C). Indeed, our results suggest that IL-10 may warrant further investigation as the agent of choice for reinforcing the tolerogenicity of ipDCs expressing CD141, either alone or in combination with a low dose of VD_3_, proposed as a conditioning regimen in forthcoming clinical trials of moDCs for the modulation of allograft rejection (8).

## Potential Pitfalls and Artifacts

While the advent of induced pluripotency provides an inexhaustible source of rare and inaccessible cell types with therapeutic potential, it is important to recognize the various drawbacks to their use. First, all cell types differentiated from pluripotent stem cells display a phenotype reminiscent of the fetal or neonatal period, a finding that has confounded the therapeutic use of cell types as diverse as hepatocytes (60) and cardiomyocytes (61). The “fetal” phenotype is especially evident among cells of the hematopoietic lineage: erythrocytes, for example, systematically fail to enucleate or progress beyond the expression of fetal hemoglobin to adult isoforms (62), greatly limiting their clinical utility. DCs differentiated from human ESCs or iPSCs likewise display hallmarks of a fetal phenotype: for instance, ipDCs secrete more abundant IL-10 than moDCs (Figure 3D) and fail to secrete IL-12, except in response to a combination of the most potent immunological stimuli (Figure 3E), a phenotype they share with moDCs isolated from neonates, which have been shown to actively repress expression of the p35 subunit of IL-12 (63, 64). Human fetal DCs have likewise been shown to suppress secretion of pro-inflammatory cytokines, additionally expressing arginase-2, whose capacity to deplete local l-arginine inhibits TNF-α secretion. Such a phenotype confers on fetal DCs the ability to induce abundant Treg cells, essential for the maintenance of maternal tolerance toward the developing fetus (65).

In addition to issues related to their unconventional provenance, the phenotype of numerous cell types differentiated from iPSCs has been shown to be influenced by the “epigenetic memory” they display for the cell type of origin, which may persist for many passages (66, 67). Given that human dermal fibroblasts remain the cell type of choice for reprogramming to pluripotency, as was the case for the C15 cell line described here (35), vestiges of the gene expression profile of the source cell type may confound the phenotypic analysis of differentiated cell types. In particular, many lineage-specific markers may be expressed at lower levels than anticipated for the equivalent cell type *in vivo*, a possible explanation for the low levels of expression of CCR7 and XCR1 by CD141^+^ ipDCs (Figure 3A). Such findings emphasize the need for functional assays, such as chemotaxis, for characterization purposes (Figures 4C,D), rather than reliance on phenotype alone. This potential cause of artifacts is most evident in the context of MHC class II expression by ipDCs: given that dermal fibroblasts actively repress MHC class II expression, which is known to be epigenetically controlled (68), ipDCs differentiated from them have been shown in both mouse and man to express these molecules at unconventionally low levels (30, 69), albeit remaining responsive to maturation stimuli and at sufficient levels to fulfill their function as professional antigen presenting cells.

Together, vestiges of a fetal phenotype and the epigenetic memory of iPSCs suggest that few, if any, cell types differentiated from iPSCs are identical to their *in vivo* counterparts. The advent of single-cell RNA-seq that has proven such a powerful technique for clarifying lineage relationships between cell types of hematopoietic origin (70), may help to further illuminate the extent of similarity or difference between CD141^+^ ipDCs and the conventional CD141^+^ subset *in vivo* and determine whether a greater allegiance to the CD14^+^ CD141^+^ subset of “regulatory” DCs described by Chu and colleagues (23) can be substantiated at the level of gene expression. While such caveats are important constraints when exploiting iPSCs to probe the molecular biology of precisely defined cell types through genome editing of the parent cell line (33), it need not undermine the therapeutic potential of the DCs differentiated from them which rely wholly on their functional capacity.

## Ethics Statement

Experiments reported in this study were carried out in accordance with the recommendations of the NRES Committee South Central—Oxford C (REC Reference: 09/H0606/5+5) following the receipt of written informed consent from all subjects in accordance with the Declaration of Helsinki.

## Author Contributions

PF conceptualized, planned, and supervised the study. PS, AL, and TD performed the experiments and analyzed the data. PS, AL, and PF wrote the manuscript which was approved by TD.

## Conflict of Interest Statement

The authors declare that the research was conducted in the absence of any commercial or financial relationships that could be construed as creating a potential conflict of interests. The derivation and use of CD141^+^ DCs from human iPSCs is the subject of a patent application (WO2012/12720601) on which PF is a named inventor.
